# Impaired Cell Viability and Functionality of Hepatocytes After Incubation With Septic Plasma—Results of a Second Prospective Biosensor Study

**DOI:** 10.3389/fimmu.2018.01448

**Published:** 2018-06-25

**Authors:** Martin Sauer, Cristof Haubner, Georg Richter, Johannes Ehler, Thomas Mencke, Steffen Mitzner, Stefan Margraf, Jens Altrichter, Sandra Doß, Gabriele Nöldge-Schomburg

**Affiliations:** ^1^Department of Anesthesiology and Intensive Care Medicine, University Hospital of Rostock, Rostock, Germany; ^2^Extracorporeal Immunomodulation (EXIM), Fraunhofer Institute for Cell Therapy and Immunology, Rostock, Germany; ^3^Division of Nephrology, Department of Medicine, University Hospital of Rostock, Rostock, Germany

**Keywords:** biosensing techniques, cytotoxicity, hepatocytes, inflammation, liver failure

## Abstract

Liver dysfunction (LD) and liver failure are associated with poor outcome in critically ill patients. In patients with severe sepsis or septic shock, LD occurred in nearly 19% of patients. An early diagnosis of LD at time of initial damage of the liver can lead to a better prognosis of these patients because an early start of therapy is possible. We performed a second prospective study with septic patients to test a new cell-based cytotoxicity device (biosensor) to evaluate clinical relevance for early diagnosis of LD and prognostic capacity. In the clinical study, 99 intensive care unit patients were included in two groups. From the patients of the septic group (*n* = 51, SG), and the control (non-septic) group [*n* = 49, control group (CG)] were drawn 20 ml blood at inclusion, after 3, and 7 days for testing with the biosensor. Patients’ data were recorded for hospital survival, organ function, and demographic data, illness severity [acute physiology and chronic health evaluation (APACHE) II-, sepsis-related organ failure assessment (SOFA) scores], cytokines, circulating-free deoxyribonucleic acid/neutrophil-derived extracellular traps (cf-DNA/NETs), microbiological results, and pre-morbidity. For the developed cytotoxicity test, the human liver cell line HepG2/C3A was used. Patients’ plasma was incubated in a microtiter plate assay with the test cells and after 6 days incubation the viability (trypan blue staining, XTT-test) and functionality (synthesis of albumin, cytochrome 1A2 activity) was analyzed. An impairment of viability and functionality of test cells was only seen in the SG compared with the CG. The plasma of non-survivors in the SG led to a more pronounced impairment of test cells than the plasma of survivors at inclusion. In addition, the levels of cf-DNA/NETs were significantly higher in the SG at inclusion, after 3, and after 7 days compared with the CG. The SG showed an in-hospital mortality of 24% and the values of bilirubin, APACHE II-, and SOFA scores were markedly higher at inclusion than in the CG. Hepatotoxicity of septic plasma was already detected with the liver cell-based biosensor at inclusion and also in the course of disease. The biosensor may be a tool for early diagnosis of LD in septic patients and may have prognostic relevance.

## Introduction

The development of liver dysfunction (LD) and liver failure in intensive care unit (ICU) patients have a relatively high incidence of 11% in all ICU patients and over 19% in patient with septic shock and is associated with increased in-hospital mortality (1–4).

Because physiological and online parameters are unable to diagnose LD early, laboratory parameters, like transaminases, albumin, and coagulation factors are commonly used, however, without convincing clinical data for detection of early LD (5). In addition, serum bilirubin is often utilized for diagnosis of (early) LD in critically ill patients (3); although an increase of bilirubin is seen late in patients, 2–3 days after initial impairment of the liver and other organ damages as displayed in sepsis-related organ failure assessment (SOFA)-, and SAPS scores (6).

LD, however, occurs as an early organ dysfunction in severe ill patients, e.g., in septic patients (3, 7). Experimental and clinical investigations have shown that impaired biliary secretion is the main component of early LD in systemic inflammatory response syndrome and sepsis (8–12).

Hepatotoxicity of inflammatory mediators like nitric oxide, chemokines and cytokines, endogen and exogen toxins like lipopolysaccharides, plasma cascade factors, and hepatic ischemia are the main pathophysiological factors for the development of LD leading to hyperbilirubinemia and intrahepatic cholestasis (3, 5, 10, 12–18).

In addition, activated neutrophils in response to infectious stimuli casting out their deoxyribonucleic acid DNA as main part of neutrophil-derived extracellular traps (NETs); so-called circulating-free deoxyribonucleic acid/neutrophil (derived) extracellular traps [cf-DNA/NETs; (19)]. NETs are emergency first-line defense mechanisms and kill microbiological pathogens in blood (19). Then again, high levels of NETs seem to be linked to multiorgan failure and sepsis (19, 20). Overwhelming NETs formation resulted in impaired microcirculation and organ damage (19).

To verify the clinical relevance, especially for (early) diagnosis of LD of a new cell-based test device [biosensor; (21)], we conducted a second prospective study with septic patients including cytokines-, and cf-DNA/NETs measurement. In a smaller first study, we showed that plasma of septic patients caused an impairment of functionality of hepatocytes in the cytotoxicity test compared with postoperative non-septic controls and healthy volunteers (22). The biosensor was actually also used for therapy monitoring of LD and liver failure in critically ill patients (23), for evaluation of experimental models of liver failure, and monitoring of hepatotoxicity of drugs and procalcitonin (PCT) (24–27).

## Materials and Methods

### Subjects and Procedures

Approval for the study from the responsible ethics committee (University of Rostock; II HV 16/2005) was obtained and for all included patients written informed consent was received. Furthermore, the study was carried out under the principles of the Declaration of Helsinki and good clinical practice.

Between June 2005 and May 2008, 51 septic patients were included in the study after screening in the two-perioperative ICUs of the University Hospital of Rostock for fulfilling the criteria of septic shock or severe sepsis (28). Organ dysfunction was defined according to the criteria of the PROWESS study (29); bilirubin levels >34.2 µmol/l (2 mg/dl) for at least 48 h was the criteria for LD (2, 3). The exclusion criteria were pre-existing liver disease, age under 18 years, pregnancy, HIV infection, and participation in another study. The control group (CG, *n* = 48) was included postoperative patients without signs of sepsis, without pre-described liver disease, and an ICU stay longer than 24 h were included.

From each patient, 20 ml blood was obtained for testing with the biosensor and screening for blood parameters, cytokines, and circulating-free deoxyribonucleic acid/neutrophil-derived extracellular traps (cf-DNA/NETs) at inclusion, after 3, and after 7 days. Patients were followed up to assess hospital survival and organ function; demographic data, illness severity, SOFA–acute physiology and chronic health evaluation (APACHE)-II, cytokines, microbiological results, and pre-morbidity were documented (Table 1).

**Table 1:** Laboratory parameters and results of APACHE II-, and SOFA scores at inclusion, after 3, and 7 days in the septic- (SG, *n* = 51), septic survived- (SSG, *n* = 39), septic non-survived- (SNSG, *n* = 12), and CG (*n* = 48); (median/0.25–0.75 quartile).

Values	At inclusion				After 3 days				After 7 days			
	Sepsis group (*n* = 51)			CG (*n* = 48)	Sepsis group (*n* = 51)			CG (*n* = 48)	Sepsis group (*n* = 51)			CG (*n* = 48)
	SG (all)	SSG	SNSG		SG (all)	SSG	SNSG		SG (all)	SSG	SNSG	
Lactate (mmol/l)	1.7 (1.5–2.9)*	1.7 (1.5–2.5)*	2.4 (1.4–5.3)*	1.1 (0.9–1.3)	1.7 (1.2–2.4)*	1.6 (1.2–2.2)*	1.9 (1.8–3.4)*	1.3 (0.9–1.6)	1.4 (1.2–2.1)	1.4 (1.1–2.0)	2.2 (1.4–3.2)	1.6 (1.3–2.8)
Bilirubin (μmol/l)	19.9 (12.1–36.8)*	19.2 (13.9–37.1)*	21.4 (11.7–33)	15.2 (11.3–18.8)	15.7 (10.7–30.9)*	15.0 (10.8–30.0)*	21.5 (10.0–35.9)*	11.0 (8.6–15.7)	13.4 (9.1–24.3)*	11.9 (8.6–23.9)*	16.8 (12.3–27.8)*	9.1 (7.6–10.8)
Ammonia (mmol/l)	51.3 (34.5–63.9)*	43.1 (30.9–60.4)*	54.4 (43.0–68.7)*	31.7 (22.4–39.4)	49.3 (38.4–65.4)*	48.1 (38.7–60.2)*	50.4 (35.5–78.8)*	31.8 (25.5–38.7)	43.7 (37.3–59.2)*	43.6 (36.8–59.8)*	48.4 (38.3–80.2)*	32.4 (24.0–43.5)
Creatinine (μmol/l)	136 (94–202)*	130 (93–193)*	161 (103–245)*	78 (66–94)	130 (92–215)*	127 (92–190)*	148 (108–232)*	77 (65–88)	83 (64–165)*	82 (62–155)	95 (82–249)*	79 (65–89)
Urea (mmol/l)	10.5 (7.3–17.4)*	9.4 (7.3–14.5)*	17.3 (7.7–22.7)*	4.4 (3.4–5.8)	12.2 (7.9–18.1)*	10.8 (7.0–15.1)*	18 (11.4–26.7)*	4.6 (3.3–5.5)	11.3 (6.3–19.7)*	11 (6.1–16.2)*	18.6 (8.2–35.0)*	4.5 (3.6–5.4)
PCT (ng/ml)	12.2 (3.9–32.5)*	10.5 (3.3–28.8)*	18.2 (4.4–47.2)*	0.5 (0.2–0.8)	5.0 (2.0–15.7)*	4.6 (2.0–12.5)*	6.9 (2.2–24.0)*	0.4 (0.1–0.6)	0.7 (0.4–3.3)*	0.7 (0.4–2.2)*	4.6 (0.4–6.7)*	0.2 (0.1–0.2)
Leukocytes (GpT/l)	14.8 (9.1–23.0)*	14.6 (8.2–22.1)*	20.2 (11.8–51.5)*	9.1 (7.6–11.7)	12.6 (8.6–16.7)*	11.6 (8.3–15.4)*	14.6 (10.5–24.4)*	8.9 (7.3–11.5)	13.3 (11.0–18.3)*	13.2 (10.4–17.7)*	15.3 (12.4–25.3)*	9.0 (8.0–10.9)
Thrombocytes (GpT/l)	191 (106–254)	201 (107–254)	174 (65–248)	184 (136–238)	170 (100–244)	187 (109–256)	149 (71–189)*	187 (149–252)	229 (135–350)*	272 (157–369)*	184 (113–236)*	334 (238–431)
Prothrombin time (%)	71 (61–82)*	73 (61–86)*	66 (61–75)*	90 (80–95)	81 (70–98)*	84 (72–102)*	65 (50–80)*	102 (92–112)	88 (74–99)*	88 (76–101)*	84 (64–96)*	99 (89–113)
APACHE II	32 (26–36)*	30 (25–35)*	36 (28–42)*	9 (7–12)								
SOFA	13 (11–15)*	12 (10–14)*	15 (12–16)*	2 (0–4)	11 (9–14)*	10 (8–13)*	13 (11–17)*	1 (0–2)	9 (3–12)*	7 (3–11)*	13 (9–17)*	0 (0–0)

*p < 0.05 versus CG.

*APACHE, acute physiology and chronic health evaluation; PCT, procalcitonin; SOFA, sepsis-related organ failure assessment; CG, control group*.

### Cell Cultures and Biosensor Methods

For the hepatocytes-based cytotoxicity assay, the human hepatocyte cell line HepG2/C3A (American Type Culture Collection CRL-10741) was used. The cells were cultivated at 37°C in a 5% CO_2_ humidified incubator with Dulbecco’s modified Eagle’s medium (GIBCO Life Technologies, Eggenstein, Germany), 10% fetal bovine serum (FBS, PAA Laboratories, Pasching, Germany), 1% antibiotics solution (penicillin G: 10,000 IE/ml/streptomycin: 10 mg/ml; PAA), and 1% 200 mM l-glutamine (PAA). Cell concentrations and vitality were determined by trypan blue (0.4%; Sigma, Seelze, Germany) staining technique using a C-Chip Neubauer improved hemocytometer (peqlab, Erlangen, Germany).

For testing the hepatotoxicity of patients’ plasma, the cells were seeded in 24-well microtiter plates in a density of 250,000 cells/well. Then, the cells were incubated for 3 days with 1 ml heparinized plasma from the subjects following a 3-day incubation period with fresh medium (1 ml). Cells and cell culture supernatants were obtained for the measurement of viability (XTT-test: dehydrogenases activity in the mitochondria, trypan blue staining: cell-count and vitality), cytochrome 1A2 activity, and synthesis of albumin. All test batches from test subjects and measurements were taken twice and a medium control was added.

The test of metabolism of ethoxyresorufine (Molecular Probes, Eugene, OR, USA) to resorufine was used for measurement of the activity of cytochrome P450 1A2; following the protocol of Kelly and Sussman (30). Resorufine concentration in the supernatants was determined at 530 nm (excitation), and 584 nm (emission) using a fluorescence multiwell plate reader (Fluoroskan Ascent Lab Systems, Vienna, VA, USA). Concentrations were estimated against a resorufine standard curve.

Albumin was measured from cell culture medium supernatant, carried out by a nephelometrical method (Immage 800, Beckman Coulter GmbH, Krefeld, Germany).

The XTT-test (Roche Diagnostics GmbH, Mannheim, Germany) was used following the describing of Scudiero et al. (31). At the start of XTT-determination 2 × 100 µl cell suspension as duplicate were transferred to a transparent 96-well plate. After adding 100 µl XTT-reaction reagent per well the absorbance of formazan was read at a wavelength of 450 nm on a microplate reader (Anthos Reader 2001, Anthos Labtec Instruments, Austria) after 1 h.

### Cytokines and cfDNA/NETs Measurement

Interleukin (IL)-1 beta, IL-6, IL-10, and tumor necrosis factor (TNF)-alpha were measured in patients’ serum with commercial ELISA kits as described by the supplier (BioSource International, Camarillo, CA, USA).

The quantification of cf-DNA/NETs was performed with a fluorescent assay (Leukocare AG, Munich, Germany). A green fluorescent dye binds DNA and the intensity of fluorescence (emission at 530 nm wavelength; Fusion, PerkinElmer, Monza, Italy) correlates with the amounts of DNA. The measurement range was between 50 and 3,000 ng/ml (20, 32). A calibration curve was conducted with a defined calf thymus DNA (Sigma, Taufkirchen, Germany) in all measurements. Former studies have shown that healthy volunteers had cf-DNA/NETs levels less than 150 ng/ml (20).

### Statistical Analysis

The results are expressed as the median with 0.25–0.75 quartile and are displayed as box plots in the figures. Nonparametric analyses were used after (negative) testing of normal distribution (with the Kolmogorov–Smirnov test; SPSS, Chicago, IL, USA). Statistical significance was analyzed with the Kruskal–Wallis one-way, the two-tailed Mann–Whitney *U*-test, the Friedman-test, and the Wilcoxon-test. Correlations between different parameters were tested with the Spearman’s-rho test. Statistical significance was assumed when the *p*-value was <0.05.

## Results

### Survival, Organ Functions, Laboratory Parameters, and Clinical Characteristics of Patients

The in-hospital mortality of the septic group (SG) was 23.5% (between day 3 and 20, *n* = 51). Two patients of the CG died in the hospital (4%, *n* = 48). All patients were surgical patients with the exception of three patients in the SG. In general, the patients of the SG fulfilled the criteria of septic shock; only 5.9% had severe sepsis. The septic patients were included in the study on average 0.5 ± 0.8 (0.2/0.8) days after beginning of severe sepsis or septic shock. In the SG, the median age (years) was 63.4 (53.2/72.9) and in the CG 68.3 (61.9/68.9); 24% of the SG- and 31% of the CG-patients were female.

Summaries of laboratory parameters and the results of the APACHE II-, and SOFA scores at inclusion, on days 3, and 7 of the CG, and the SG are displayed in Table 1. In the SG, the APACHE II was 32 at inclusion (CG: 9). The SOFA scores and the values of bilirubin, lactate, ammonia, creatinine, urea, PCT, leukocytes, and prothrombin time differed significantly between the SG and the CG at inclusion, and on day 3; the non-survivors of the SG had more pathologically values than the survivors.

Twenty-five patients of the SG (49%) developed acute kidney injury, and 31.4% (16 patients) needed renal replacement therapy (continuous methods). Criteria for LD (2, 3) were fulfilled in 17 patients (33.3%) of the SG at inclusion, in 16 patients (31.4%) after 3 days, and in 7 patients (13.7%) after 7 days. Five patients with LD at inclusion died during the hospital stay.

The sources of primary infection and results of microbiological analysis in the SG are displayed in Table 2. The predominant sources of infection were peritonitis, wound infections, and pneumonia; in only 68.6% of septic patients, the microbiological tests provided valuable findings.

**Table 2:** Source of primary infection and results of microbiological analysis in the septic group (SG, *n* = 51).

Source of primary infection	Peritonitis	Wound-infection/abscess	Pneumonia	Urogenital infection
Patients (*n*)	24	7	20	1

**Microbiological results**	**Wound-/intraoperative-swap**	**Blood culture**	**Bronchial lavage/tracheal secrete**	**Urine-culture**

Fungi (*n*)	11	1	0	0
Gram-positive bacteria (*n*)	10	3	1	1
Gram-negative bacteria (*n*)	11	3	12	2

### Cytokines and cfDNA/NETs Measurement

All values of IL-1-beta, TNF-alpha, and IL-10 were below 21 pg/ml (as median) in the SG and CG (Table 3). Higher levels of IL-6 were found in the SG; interestingly, the survivors had higher values of IL-6 than the non-survivors. A significant decrease of IL-6 and TNF-alpha was only seen in the SG between inclusion and day 3.

**Table 3:** Cytokines values at inclusion, and after 3 days in the septic- (SG, *n* = 51), septic survived- (SSG, *n* = 39), septic none survived- (SNSG, *n* = 12), and CG (*n* = 48); (median/0.25–0.75 quartile).

Cytokine (pg/ml)	At inclusion				After 3 days			
	Sepsis group (*n* = 51)			CG (*n* = 48)	Sepsis group (*n* = 51)			CG (*n* = 48)
	SG (all)	SSG	SNSG		SG (all)	SSG	SNSG	
IL-1 beta	3.9 (2.4–4.5)	5.8 (2.5–6.5)	2.7 (2.5–2.9)	2.5 (2.4–2.6)	2.5 (2.3–2.6)	2.6 (2.4–2.8)	2.4 (2.2–2.9)	2.6 (2.5–2.7)
IL-6	268 (106–557.5)^+,°^	268 (116.5–557.5)^#,°^	226 (98–501.5)^§,°^	78 (42.1–143)^°^	45.3 (25.9–90.5)	48.2 (27–89)	42.3 (25.4–155.9)	30.5 (22.1–58)
IL-10	11.1 (2.4–25.9)^+^	12.3 (3.8–24.1)^#,°^	4.6 (2.5–33.3)	2.5 (2.3–8.6)	7.5 (2.4–12)^+^	7.2 (2.3–11.9)^#^	7.9 (2.5–17.5)^§^	2.5 (2.2–2.6)
TNF-alpha	20.4 (12.9–32.2)^+,°^	20.4 (13.9–29.8)^#,°^	20.7 (11.2–41.9)^§,°^	7.7 (5.7–9.7)	14.7 (10.2–19.9)^+^	15.5 (11.2–19.9)^#^	10.8 (7.1–23.2)	6.6 (5.6–9.7)

*Statistically significant (*p* < 0.05): ^#^between SSG and CG (*U*-test); ^§^between SNSG and CG (*U*-test); ^+^between SG and CG; ^°^between inclusion and day 3*.

*IL, interleukin; TNF, tumor necrosis factor; CG, control group*.

**Figure 1:**
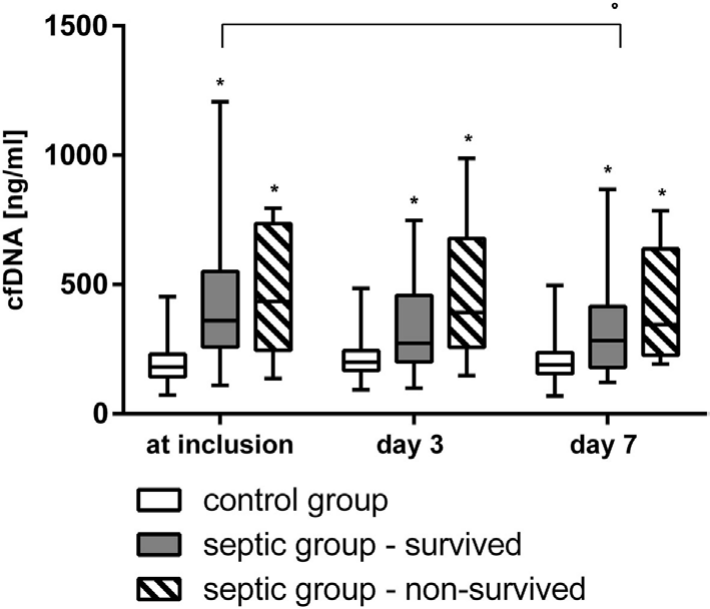
The values of circulating-free deoxyribonucleic acid (cf-DNA, median/0.25–0.75 quartile) at inclusion, after 3, and 7 days in the survivors (*n* = 39) and the non-survivors (*n* = 12) of the septic group and in the non-septic control group (CG) (*n* = 48). *p < 0.05 versus CG (Mann–Whitney *U*-test). °*p* < 0.05 between inclusion and day 7 (Wilcoxon-test).

The values of cf-DNA/NETs were significantly higher in the SG than in the CG at inclusion, after 3, and after 7 days (Figure 1). A significant decrease of cf-DNA/NETs levels from inclusion to day 7 was observed in the survivors of the SG, but not in the non-survivors and in the CG.

### Results of the Hepatocyte-Based Cytotoxicity Tests

**Figure 2:**
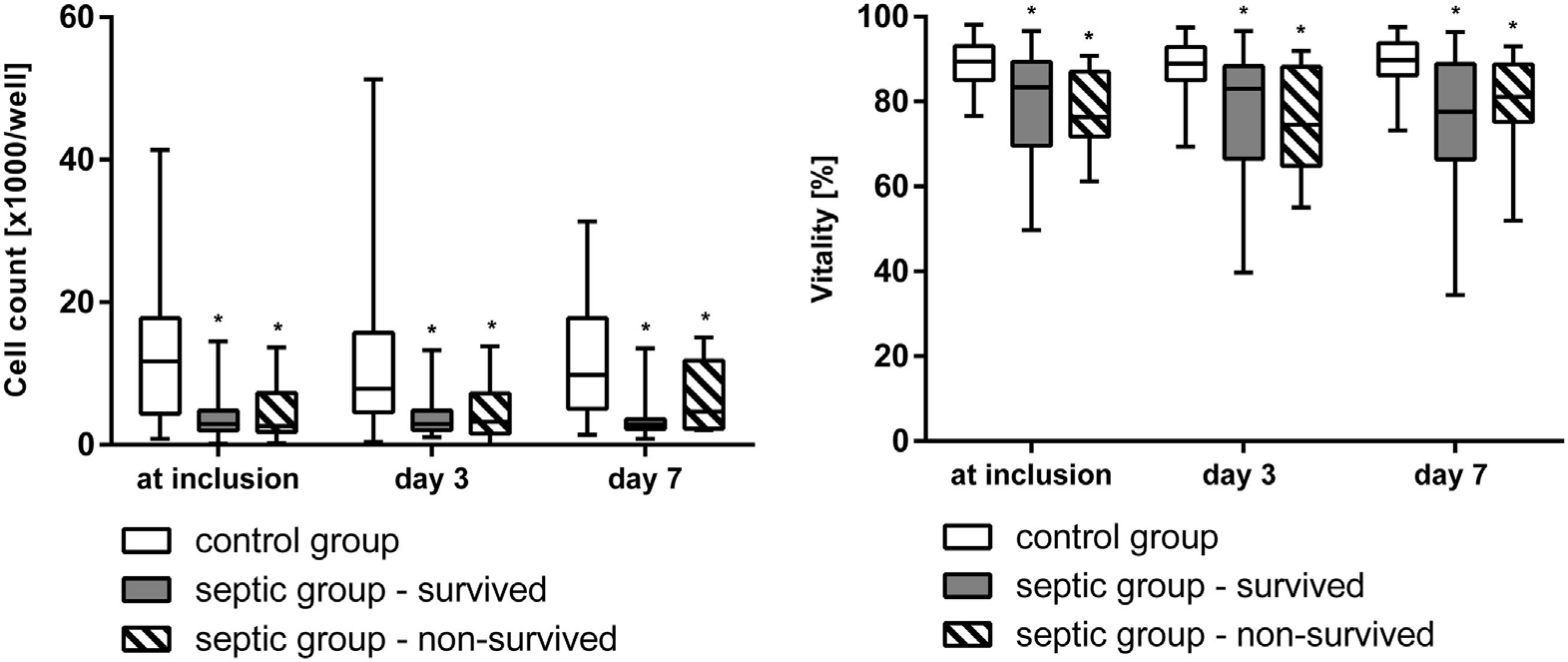
Cell count and vitality (trypan blue staining, median/0.25–0.75 quartile) of HepG2/C3A cells incubated with plasma from survived and non-survived septic patients and non-septic control patients at inclusion, after 3, and 7 days. *p < 0.05 versus control group (Mann–Whitney *U*-test).

**Figure 3:**
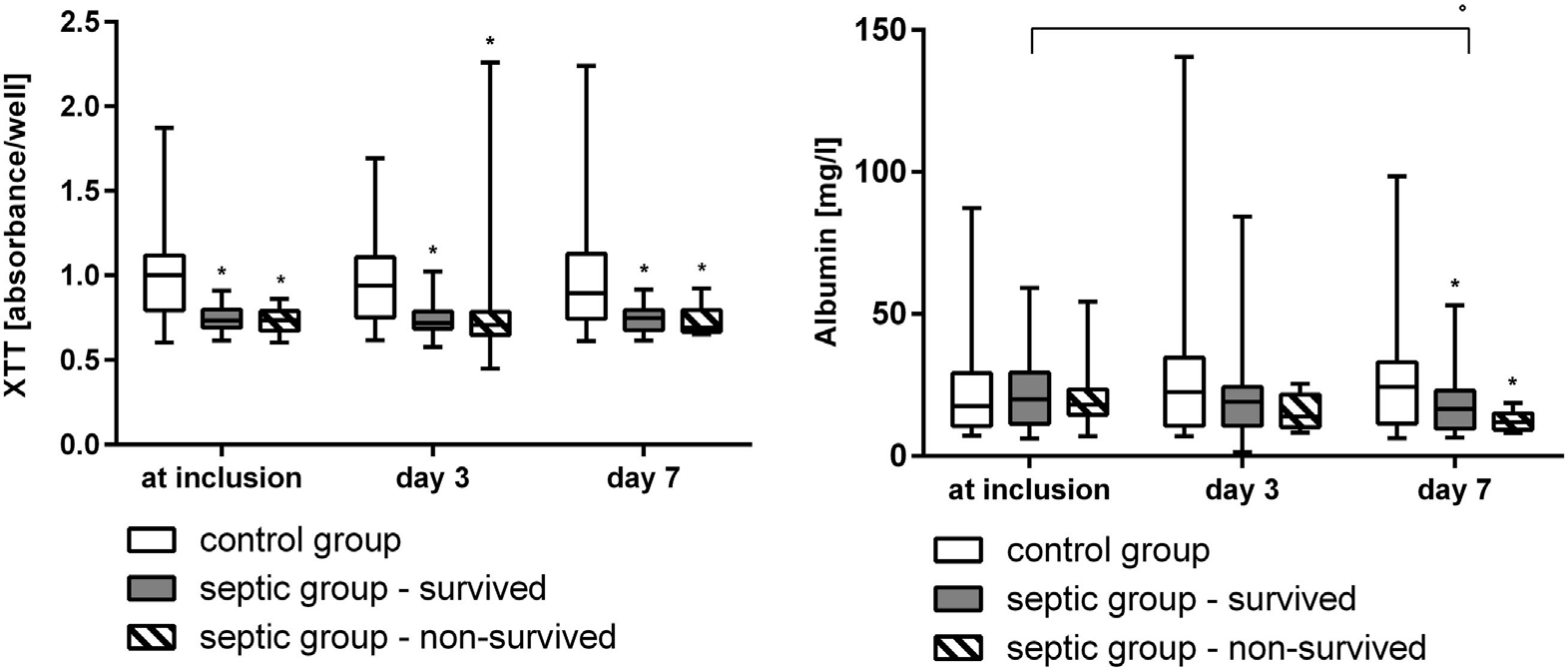
Results of the XTT-test (dehydrogenases activity in the mitochondria) and albumin synthesis of HepG2/C3A cells incubated with plasma from survived and non-survived septic patients and non-septic control patients at inclusion, after 3, and 7 days displayed as median/0.25–0.75 quartile. *p < 0.05 versus control group (Mann–Whitney *U*-test). °*p* < 0.05 between inclusion and day 7 (Wilcoxon-test).

**Figure 4:**
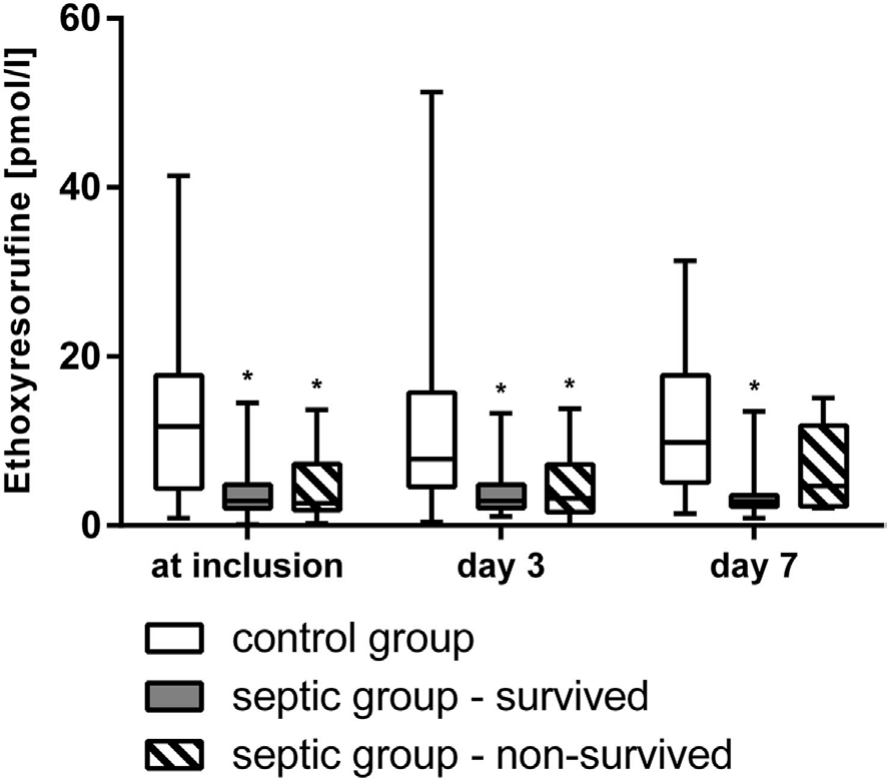
Metabolism of ethoxyresorufine to resorufine (activity of cytochrome 1A2, median/0.25–0.75 quartile) of HepG2/C3A cells incubated with plasma from survived and non-survived septic patients and non-septic control patients at inclusion, after 3, and 7 days. *p < 0.05 versus control group (Mann–Whitney *U*-test).

At inclusion, after 3, and 7 days the vitality and the cell count (Figure 2), the activity of mitochondrial dehydrogenase (XTT-test, Figure 3), and the metabolism of ethoxyresorufine (cytochrome 1A2 activity, Figure 4) were significantly decreased in the SG, compared to the test results of the CG. These impairments of viability and function of test cells were more pronounced in non-survivors of the SG compared with survivors only at inclusion (Figures 2–4). By contrast, the synthesis of albumin was impaired later and also pronounced in non-survivors of the SG after 3 and 7 days in SG compared to the CG (Figure 3).

### Correlation Analysis at Inclusion

Correlations between the hepatocyte-based cytotoxicity test parameters with bilirubin, alanine aminotransferase (ALAT), asparagine aminotransferase (ASAT), and ammonia were not observed. Significant negative correlations were found between the cytochrome 1A2 activity, the XTT-test, the vitality, and the cell count with the APACHE II-, and SOFA scores, lactate, and PCT (ρ between −0.3 and −0.5, *p* < 0.05). In addition, correlations were observed between the cytochrome 1A2 activity with TNF-alpha, IL-6, and IL-10 (ρ between −0.3 and −0.4, *p* < 0.001); the levels of TNF-alpha also correlated with the results of the XTT-test and the vitality (ρ = −0.3, *p* < 0.01).

The values of bilirubin correlated with the APACHE II-, SOFA scores, and lactate (ρ = +0.3, *p* < 0.01).

Significant correlations were found between the values of cf-DNA/NETs with the APACHE II-, and SOFA scores, and PCT (ρ between +0.4 and +0.6, *p* < 0.001) and with the cytochrome 1A2 activity, the XTT-test, the vitality, and the cell count (ρ between −0.3 and −0.5, *p* < 0.05).

The measured cytokines did not correlate with ALAT, ASAT, and ammonia; however, the cytokines IL-6, IL-10, and TNF-alpha correlated with bilirubin (ρ between +0.2 and +0.4, *p* < 0.05), PCT (ρ between +0.4 and +0.6, *p* < 0.001), lactate (ρ between +0.3 and +0.5, *p* < 0.01), APACHE II- (ρ between +0.4 and +0.6, *p* < 0.001), and SOFA- (ρ between +0.4 and +0.7, *p* < 0.001) scores.

## Discussion

### Clinical Characteristics of Study Cohort, Outcome, and Serum Bilirubin

In this study, we included 51 patients in general septic shock and observed a relatively low in-hospital mortality of 23.5% in comparison to other studies (33, 34). The time point of inclusion in the study after the beginning of septic shock was early in our mainly surgical patient cohort.

Liver dysfunction or liver failure occurred in 33.3% (*n* = 17) of the patients in the SG at inclusion; five patients with LD at inclusion died during the hospital stay. The rate of LD was much higher in our septic study cohort in comparison to other studies (3, 4). Interestingly, the seven other non-survivors in the SG did not show an increase of bilirubin at inclusion and we found only low correlations between morbidity and degree of multiorgan failure as displayed by the APACHE-II-, SOFA scores, and values of lactate with the results of serum bilirubin.

### Hepatocyte-Based Cytotoxicity Tests and Role of Cytokines

In the state of severe sepsis or septic shock, the functionality of hepatocytes is partly decreased (5). The aim of this study was to test the direct hepatotoxicity of plasma from septic patients in a standardized hepatocyte-based cytotoxicity test in a second biosensor study (24–27).

In this study, we saw an impairment of viability and functionality of test cells after incubation with plasma from patients in general septic shock. The cytochrome 1A2 activity, the XTT values (activity of mitochondrial dehydrogenases), the cell count, and the vitality were significantly lower in the SG compared to the CG at inclusion, after 3, and after 7 days. In addition, the plasma of non-survivors in the SG led to a more pronounced impairment of test cells than the plasma of survivors at inclusion. These results support the aim of the study that the cell-based biosensor used may be a tool of early diagnosis for LD and has prognostic value. In addition to this thesis, we found significant negative correlation between the cytochrome 1A2 activity, the XTT-test, the vitality, and the cell count with the APACHE II- and SOFA scores, lactate, and PCT at inclusion. By contrast, the albumin synthesis in test cells seems to be a late changing parameter and is not valuable for early detection of LD.

In this study, correlations between static liver markers like bilirubin with the results of the hepatocyte cytotoxicity test parameters at inclusion were not seen. This may be due to the fact that bilirubin and other classical liver parameters increase late in LD or liver failure, especially in septic patients (6).

The impairment of cell function and viability seen in HepG2/C3A after incubation with septic plasma can be caused by endogenous and exogenous toxins, drugs, and metabolites (15, 35, 36). In addition, pro-inflammatory and anti-inflammatory cytokines modulate and impair the function of hepatocytes. We tested the patients’ plasma at inclusion, and after 3 days for IL-1 beta, TNF alpha, IL-6, and IL-10. The values of measured cytokines were relatively low, but higher in the SG than in the CG; only the values of IL-6 were markedly increased at inclusion in the SG but decreased after day 3.

Due to relative low values of cytokines with exception of IL-6, we observed only a few correlations between cytokines and parameters of the biosensor: between the cytochrome 1A2 activity with TNF-alpha, IL-6, and IL-10 at inclusion and results of the XTT-test, and the vitality with the levels of TNF-alpha also at inclusion.

Many cytokines, mainly the pro-inflammatory cytokines TNF-alpha, IL-1 beta, and IL-6, cause an impairment or dysregulation of the viability, the function, and apoptosis of human hepatocytes and hepatocyte cell lines, e.g., in HepG2/C3A cells [for review, see Ref. (35–48)]. These impairments of hepatocytes lead to dysfunction of mitochondria, to a decreased level of negative acute phase proteins like albumin, and a decreased activity of some P450 cytochromes including CYP 1A2 (35, 36, 40, 49). The lower values of biosensor parameters found in the cells incubated with septic plasma may be explained partly by the effects of pro-inflammatory cytokines on the sensor cells.

For our hepatocyte-based cytotoxicity test, we worked with the well-characterized cell line HepG2/C3A (50). The cell line is commonly used for toxicological studies (51, 52) and shows many functionalities after stimulation comparable with normal human hepatocytes [for review, see Ref. (30, 44, 48, 50, 53–60)]. Therefore, the HepG2/C3A cell line is a source for bioartficial liver support systems (61).

In a previous work from our research group, the testing of antimycotics (caspofungin, anidulafungin, and fluconazole) with HepG2/C3A cells compared with human primary isolated hepatocytes provided similar results regarding cytochrome 1A2 activity, vitality, and activity of mitochondrial dehydrogenase [for review, see Ref. (24)].

### Role of cf-DNA/NETs in Sepsis and Liver Failure

NETs play a diametrical role in sepsis (19). As part of the innate immune system NETs quickly trap and neutralize microbes in tissues and blood (19). Then again, NETs also lead to damage of inflamed tissues as carrier of molecules with autodestructive immune effector functions, e.g., extracellular DNA, elastase, myeloperoxidase, lactoferrin, pentraxin, and bactericidal/permeability-increasing protein (19). During early stage sepsis, activated neutrophils traps and accumulate primarily in the sinusoids of the liver (38). The NETs in the liver sinusoids cause tissue- and endothelial damage resulting in denuding of the endothelium and allows platelets to enter the space of Disse (37). These mechanisms lead to extravasated platelet aggregation. NETs and platelet aggregation result in thrombosis and impaired blood flow in the liver sinusoids and seem to be an important cause of LD in sepsis (37).

In line with results of former studies (19, 20, 39), the values of NETs were increased in the septic patients, more pronounced in non-survivors and compared with the non-septic control patients in our study. We also saw correlations between morbidity and degree of multiorgan failure displayed in the APACHE-II, and SOFA scores and the values of cf-DNA/NETs in concordance with other studies (20, 39). Interestingly, we could also observe correlations of some parameters of the biosensor and the values of the cf-DNA/NETs at inclusion that may support the diagnostic capacity of the biosensor for LD in sepsis.

### Limitations of the Study and Summary

The test time with the hepatocyte-based biosensor of 3–6 days in this study is not suitable for early diagnosis of LD. However, by optimization of the biosensor, the incubation time has meanwhile been able to have reduced to 20 h (unpublished data). By technical improvements of the cell culture system (shaking instead of a resting system) and the increase of the concentration of FBS in the cell culture medium, we achieved comparable viability and functionality of sensor cells in human plasma after incubation times of 6 days and of 20 h. The reduction of test time is an important condition for usability in clinical practice (Appendix).

Plasma of healthy volunteers was not tested in the presented analysis. In our former study, the values of the hepatocyte-based test were comparable and without significant difference between healthy volunteers and the postoperative non-septic CG (22).

In conclusion, hepatotoxicity of septic plasma was already detected with the liver cell-based cytotoxicity at inclusion and also in the course of disease. The causes of these cellular impairments need further basic science and clinical investigations. The influence of NETs on the development of liver failure in septic patients seems to be an interesting approach. Higher levels of cf-DNA/NETs and impairments in all parameters of the hepatocyte biosensor were associated with a worse outcome in this study. Since bilirubin is a late parameter in LD and only markedly increased in advanced liver damage (5, 6), cf-DNA/NETs- and hepatocyte-based biosensoring may help to detect “subclinical” liver damage with prognostic relevance; further clinical validation, especially for usefulness as early diagnostic tools for LD, are necessary.

## Ethics Statement

The study received ethics approval from the local research ethics committee (University of Rostock; II HV 16/2005), which include compliance with the principles of good clinical practice. The study abided by the principles of the Declaration of Helsinki. Written informed consent was obtained from all subjects or from the patients’ representatives if direct consent could not be received.

## Author Contributions

MS and GN-S participated in the design of the study. MS did the regulatory work and coordinated the study; wrote the manuscript; revising work was done from all other authors. MS, GR, CH, and SD did the data analysis. MS, CH, TM, JE, GN-S, and SMitzner were clinical investigators. SMargraf and JA analyzed the clinical probes for cf-DNA/NETs.

## Conflict of Interest Statement

The authors declare that the research was conducted in the absence of any commercial or financial relationships that could be construed as a potential conflict of interest.

## Abbreviations

ALAT, alanine aminotransferase; APACHE, acute physiology and chronic health evaluation; ASAT, asparagine aminotransferase; ATCC, American Type Culture Collection; cf-DNA, circulating-free deoxyribonucleic acid; CG, control group; ICU, intensive care unit; IL, interleukin; LPS, lipopolysaccharides; NETs, neutrophil-derived extracellular traps; PCT, procalcitonin; SG, septic group; SIRS, systemic inflammatory response syndrome; SOFA, sepsis-related organ failure assessment; TNF, tumor necrosis factor.
