# Supplementary material for: Impaired Cell Viability and Functionality of Hepatocytes After Incubation With Septic Plasma—Results of a Second Prospective Biosensor Study
Source: Front Immunol. 2018 Jun 25;9:1448. doi: 10.3389/fimmu.2018.01448 (PMC6026797; doi:10.3389/fimmu.2018.01448)
Supplement: Supplementary file 1 [file data_sheet_1.docx]

**Appendix:**

**Biosensor Results in comparison to the test-time, n=24, Mean (SD), Healthy plasma**

| ***Parameter*** | **6 days** | **20 hours** |
| --- | --- | --- |
| ***Number of cells***  *(x 1000)/well* | 18 (11) | 16 (10) |
| ***Vitality***  *(%)* | 89.2 (4.2) | 94.7 (2.6) |
| ***XTT***  *(Extention /Well)* | 1.2 (0.51) | 0.93 (0.42) |
| ***Ethoxyresorufin-test***  (pmol/l) | 14.6 (9.3) | 13.8 (9.2) |
| ***Synthesis of Albumin***  *(mg/l)* | 19.9 (28.2) | 18.0 (24.4) |
